# Local resection for solid pseudopapillary neoplasms of the pancreas shows improved postoperative gastrointestinal function and reduced mental stress: a multiquestionnaire survey from a large cohort

**DOI:** 10.1097/JS9.0000000000000702

**Published:** 2023-10-11

**Authors:** Yuze Hua, Xia Hong, Menghua Dai, Jiayi Li, Sen Yang, Junchao Guo, Weibin Wang, Qiang Xu, Xianlin Han, Mengyi Wang, Nan Huang, Huaijin Zheng, Jorg Kleeff, Qiaofei Liu, Wenming Wu, Taiping Zhang, Quan Liao, Yupei Zhao

**Affiliations:** a Department of General Surgery, State Key Laboratory of Complex Severe and Rare Diseases, Peking Union Medical College Hospital, Key Laboratory of Research in Pancreatic Tumors (CAMS), Peking Union Medical College; b Department of Psychological Medicine, Peking Union Medical College Hospital, Peking Union Medical College, Chinese Academy of Medical Sciences, Dongcheng District, Beijing, People’s Republic of China; c Department of Visceral, Vascular and Endocrine Surgery, Martin-Luther-University Halle-Wittenberg, Halle (Saale), Germany

**Keywords:** gastrointestinal function, local resection, mental stress, minimally invasive surgery, solid pseudopapillary neoplasm of the pancreas

## Abstract

**Background:**

Solid pseudopapillary neoplasm (SPN) of the pancreas is a rare, low-grade malignant pancreatic tumor with a highly favorable prognosis. Most SPN patients are young and middle-aged women. The main controversial topic for SPN is local resection (LR) versus radical resection (RR). Theoretically, LR could lead to better gastrointestinal function (GIF) and less mental stress. However, no data is available to support this hypothesis.

**Methods:**

All SPN patients undergoing surgical treatment in Peking Union Medical College Hospital from 2001 to 2021 were included in the study. A cross-sectional online multiquestionnaire survey containing 110 questions was sent to them (Clinicaltrial.org, NCT05604716). This online multiquestionnaire survey focused on GIF and mental stress and consisted of eight questionnaires. Multiple linear regression analysis was conducted to identify independent factors impacting GIF and mental stress.

**Results:**

A total of 183 cases provided valid results. Among them, 46 patients (25.1%) underwent LR, and 137 (74.9%) underwent RR. Ninety-four cases (51.4%) underwent minimally invasive surgery (MIS), while 89 (48.6%) underwent open surgery. The average GSRS score of the patients was 1.9±0.7, indicating that most suffered from mild gastrointestinal dysfunction. The scores of PHQ-9 and GAD-7 in 16 patients (8.7%) and 27 (14.8%) patients, respectively, were beyond 10.0, which indicated clinical depression and anxiety. Additionally, 19 (10.4%) patients reported poor ability to work, and 31(16.9%) patients had significant body image concerns. Compared to other clinicopathological characteristics, LR (LR vs. RR: PHQ-9 score, *P*=0.018; WAI average score, *P*=0.010; EORTC QLQ-C30, nine subdomains, *P*<0.05; GSRS average score, *P*=0.006) and MIS (MIS vs. open surgery: EORTC QLQ-C30, three subdomains, *P*<0.05; GSRS average score, *P*=0.006) were the most significant factors predicting improved GIF and reduced mental stress.

**Conclusions:**

This study systematically presents postoperative GIF and mental stress of SPN patients using validated multiquestionnaires for the first time. It provides solid evidence that LR and MIS can improve GIF and reduce mental stress after surgery for SPN patients, which could be helpful for the surgeons to make more personalized surgical plans for their patients.

## Introduction

Highlights

- Solid pseudopapillary neoplasm (SPN) has extremely favorable oncological outcomes after surgery. Most patients are young or middle-aged women in their early careers. Therefore, postoperative gastrointestinal function (GIF) and mental stress of these patients are the main concerns. However, no information on GIF and the mental stress has been reported yet. We provide this information by using a multiquestionnaire survey in a 20-year cohort of SPN patients from a high-volume center.
- Local resection (LR) and radical resection have been reported to have comparable oncological outcomes for SPN patients in previous studies. However, their impact on GIF and mental stress has not been reported. We now show that local resection has improved GIF and reduced mental stress compared to radical resection.
- The results of this study can aid surgeons in making more precise and individualized plans for patients to meet the balance between oncological outcomes and mental stress and GIF.

Solid pseudopapillary neoplasm (SPN) of the pancreas is a rare pancreatic tumor comprising nearly 1–3% of all pancreatic tumors^1,2^. The incidence or detection rate of SPN has been on the rise due to the more widespread use of abdominal imaging^3–6^. The primary controversial topic surrounding SPN is the choice between local resection (LR) and radical resection (RR). In theory, LR is less invasive and could significantly preserve organ function, leading to better postoperative gastrointestinal function (GIF) and less mental stress. On the other hand, RR with lymph node dissection could achieve better oncological outcomes. Referring to the oncological outcome of SPN, numerous reports from high-volume centers have comprehensively demonstrated that the incidence of lymph node metastasis is extremely low. Consequently, LR and RR have comparable long-term oncological outcomes^3,7–10^. However, data on postoperative GIF and mental stress of SPN patients have not been reported yet, and there is no supportive evidence regarding the impact of LR and RR on these aspects. Herein, the authors adopted validated questionnaires previously used in studies of other tumors after surgery^11–13^ to assess GIF and mental stress in a large cohort of SPN patients using a novel online methodology of eight questionnaires. For the first time, this study provides information on SPN patients’ GIF and mental stress using validated questionnaires. The results of this study can assist surgeons and patients in making better, individualized clinical decisions to improve GIF and reduce mental stress after surgery.

## Patients and methods

### Patients and follow-up

This cross-sectional single-center study collected data from hospitalized SPN patients between October 2001 and April 2021 from the Peking Union Medical College Hospital electronic medical record system. The contact, demographic, and clinical-pathological information, including telephone number, age, sex, occupation, marital status, educational level, symptoms, diagnosis, surgical procedures and methods, postoperative complications, and pathological outcomes, were retrieved. All patients had a definitive pathological diagnosis of SPN. The definition and severity of complications were defined according to the International Study Group of Pancreatic Surgery (ISGPS) and the Clavien–Dindo classification^14,15^. The histopathological characteristics of the tumors included lymph node metastasis, distant metastasis, and organ and tissue invasion (fatty tissue, nerves, vessels, bile duct, duodenum, stomach, transverse colon, and others).

All patients with available telephone numbers were called and asked whether they could accept our online multiquestionnaire survey via the WeChat App. Subsequently, we obtained the responders’ WeChat accounts and sent them the super link to access the multiquestionnaire survey. Before answering the survey questions, they were required to read the electronic written consent, and only those who agreed and confirmed the consent could proceed to the survey. The online survey was conducted between November 2022 and January 2023. Participation in the survey was entirely voluntary. Finally, the raw data from the survey were downloaded and analyzed. This study was approved by the Institutional Review Board of PUMCH (SK1034) and registered at Clinicaltrial.org (NCT05604716) in November 2022.

### Online multiquestionnaire survey

Eight questionnaires evaluated GIF and mental stress. All questionnaires were electronically distributed using a personal online data collection tool. GIF was assessed via the Gastrointestinal Symptom Rating Scale (GSRS) and nine symptom scales of the European Organization for Research and Treatment of Cancer Quality of Life Questionnaire-Core 30 (EORTC QLQ-C30). The GSRS is a 15-item questionnaire that quantifies common GI symptoms, including abdominal pain, reflux, indigestion, diarrhea, and constipation, on a 7-point scale^16^. Higher scores indicate more severe symptoms and dysfunction. Although initially developed for patients with peptic ulcer disease and irritable bowel syndrome, it has been used in various gastrointestinal diseases and in postsurgical patients undergoing upper gastrointestinal surgery^17,18^. EORTC QLQ-C30 is a commonly used tool for assessing multiple mental stress domains, including function due to role, function due to emotional health, emotional well-being, social functioning, and general health. Each domain underwent a linear transformation to standardize the raw score on a scale of 0–100, with higher scores indicating less mental stress^19^. Social psychological functions are also critical components after surgery. We applied the depression scale of the Patient Health Questionnaire-9 (PHQ-9) and the General Anxiety Disorder 7-item Scale (GAD-7) to evaluate the degree of depression and anxiety. The Sense of Coherence scale (SOC-9) evaluated the patient’s vulnerability to illness. The Post-Traumatic Embitterment Disorder self-rating scale (PTED-21) presented the intensity of reactive stimulus-bound embitterment^16,20^. Patient satisfaction with certain aspects of his or her body’s appearance was scored by the Body Image Scale (BIS). The Working Ability Index (WAI) measures workers’ perceptions regarding their physical, mental, and social health and ability to cope with job demands. A brief introduction and explanation of these eight scales was presented in Table 1. The super link to the online survey was https://www.wjx.cn/vm/hnHjC1U.aspx#. The whole questionnaire included 110 questions in total. A validated response was defined as: (1) all questions of the survey were answered; (2) the participants answered the quality-control questions correctly; (3) the time to answer the questions was more than 4 min. This work has been reported in line with the strengthening the reporting of cohort, cross-sectional, and case–control studies in surgery (STROCSS) criteria^21^ (Supplemental Digital Content 1, http://links.lww.com/JS9/B185).

**Table 1:** Eight questionnaires evaluating GIF and mental stress of SPN patients.

	Scale	Content	Score range	Cutoff value
GIF	Gastrointestinal Symptoms Rating Scale (GSRS)	symptoms (abdominal pain, reflux, indigestion, diarrhea, constipation)	1–7 (average score)	NA
	European Organization for Research and Treatment of Cancer Quality of Life Questionnaire-Core 30 (EORTC QLQ-C30)	nine symptoms (fatigue, nausea, vomiting, pain, dyspnea, insomnia, appetite loss, constipation, diarrhea) and functioning subscales (financial difficulty; social, cognitive, emotional, role, physical functioning; global health)	0–100	NA
Mental stress and functions	Depression Scale of the Patient Health Questionnaire-9 (PHQ-9)	frequency of symptoms to evaluate the degree of depression	0–27	≥10: clinically diagnosable depression
	General Anxiety Disorder 7-item Scale (GAD-7)	frequency of symptoms to evaluate the degree of anxiety	0–21	≥10: clinically diagnosable anxiety
	Sense of Coherence scale (SOC-9)	vulnerability to illness	9–63	NA
	^a^Post-Traumatic Embitterment Disorder self-rating scale (PTED-21)	measures the intensity of reactive stimulus-bound embitterment	0–80	NA
	Body Image Scale (BIS)	rate the patient’s satisfaction with certain aspects of their body’s appearance	0–30	≥10: body image anxiety
	Working Ability Index (WAI)	measure perceptions of workers regarding their physical, mental, and social health and ability to cope with job demands	7–49	<27: poor 27–35: moderate 36–44: good ≥45: excellent

a Question No.14 of PTED-21 is not applicable to the survey.

### Statistical analysis

Measurement data was presented as mean±SD or median with an interquartile range, and categorical data were expressed as numbers with percentages. Measurement data was compared using the independent *t* test or analysis of variance when the data followed a normal distribution. When the measurement data did not follow a normal distribution, the nonparametric rank-sum test was adopted. Categorical data were compared using the *χ* ^2^ test.

A multivariate linear regression model was used to explore the causal relationship and to identify independent predictors of mental stress and GIF and was described using a *β* coefficient and 95% CI. Correlations between mental stress and GIF were analyzed by Spearman correlation analysis. Statistical significance was set at a two-tailed *P*-value of <0.05. Statistical analyses were performed using the SPSS statistics software package (version 26.0; IBM). The graphs were produced using GraphPad Prism [9.5.0(525)] and SPSS.

## Results

### Patient enrollment and basic information

**Figure 1:**
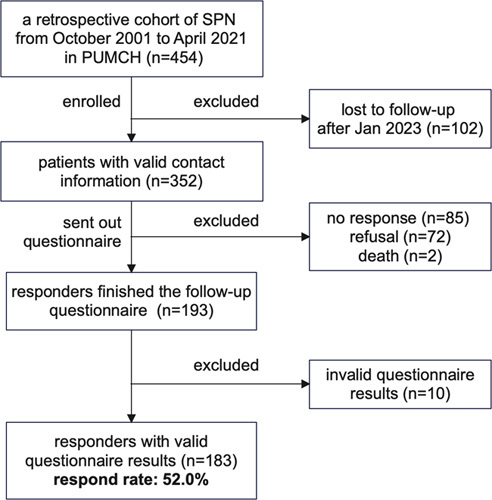
Flowchart of patient enrollment.

Information on 454 SPN patients who underwent surgery from 2001 to 2021 was retrieved. A total of 352 patients responded to our telephone-call follow-up and received the online questionnaire. Eighty-five patients did not respond, and 72 patients refused to answer. We received 193 questionnaires and 183 of those could be validated (Fig. 1). The demographic and clinical-pathological characteristics of 454 cases have been reported previously^10^. The characteristics of the included 183 patients was shown in Table 2. Among them, 138 (75.4%) were female, and 65 (35.5%) were unmarried at the time of surgery. Forty-six patients (25.1%) underwent LR, while 137 (74.9%) underwent RR. Eighty-nine patients (48.6%) had open surgery, while 94 (51.4%) had minimally invasive surgery (MIS). Of the 183 patients, 38 (20.8%) showed features of invasiveness on histopathological examination.

**Table 2:** Clinical and surgical features of patients.

Item		*n* (%)	Item		*n* (%)
Age operated	<25 years	58 (31.7)	Surgery procedure	LR	46 (25.1)
	≤25 years, <45 years	106 (57.9)		RR	137 (74.9)
	≤45 years, ≤60 years	15 (8.2)	Surgery complication (Clavien–Dindo 2 and beyond )	2	24 (13.1)
	>60 years	4 (2.2)		3	7 (3.8)
Current age	<25 years	21 (11.5)		4	2 (1.1)
				5	0 (0)
	≤25 years, <45 years	114 (62.3)			
	≤45 years, ≤60 years	40 (21.9)			
	>60 years	8 (4.4)			
Sex	Female	138 (75.4)	Surgery method	Open	89 (48.6)
	Male	45 (24.6)		MIS	94 (51.4)
Marriage operated	Unmarried	65 (35.5)	Pathology	Invasiveness	38 (20.8)
	Married	118 (64.5)		Noninvasiveness	145 (79.2)
Current marriage	Unmarried	40 (21.9)	Postsurgery interval	<36 m	33 (18)
	Married	139 (76)		≤36,≤60 m	60 (32.8)
	Divorced	4 (2.2)		>60 m	90 (49.2)
Educational level	Junior high school degree and below	88 (48.1)	Work type	Mental labor	121 (66.1)
	University and postgraduate	95 (51.9)		Physical labor	10 (5.5)
				Both	32 (17.5)
				No work	20 (10.9)

LR, local resection (enucleation, segmentation, duodenum preserving pancreatic head resection); MIS, minimally invasive surgery; RR, Radical resection (PD, PPPD, DP, DPS).

### Mental stress and GIF of the patients after surgery

**Figure 2:**
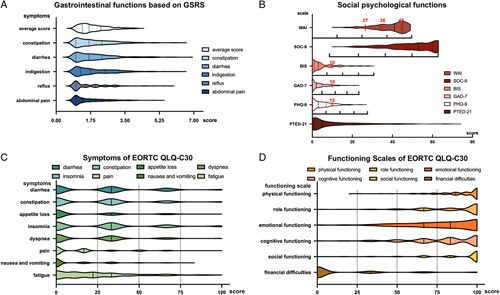
Mental stress and GIF of SPN patients. (A): Scores of GSRS: the average scores ranging from 1 to 7 represent no discomfort, minor, mild, moderate, moderately severe, severe, and very severe discomfort. (B): Scores of social psychological function: for WAI, the score less than 27 represents poor, 27–35 moderate, 36–44 good, and greater than or equal to 45 excellent ability to work. Ten is the cutoff value of BIS and a score greater than or equal to 10 means that the patient has body image anxiety. The red dotted line crossing the GAD-7 and PHQ-9 is the cutoff value for obvious clinical symptoms of anxiety and depression. The black dotted lines represent the quartiles of the number of patients. The quartile of the score range is shown below every graph. (C) Scores of symptoms and (D) scores of function of the EORTC QLQ-C30. For most patients, symptoms were mild, and about 80% of patients’ scores were under 50. The most severe general symptom was fatigue. Nearly 20% of patients had impaired function. The standard formula transformed the scores from raw to 100 through a linear transformation.

The average scores for each symptom of GRSR were above the median value of the scale (3.5) in less than 20% of the patients, suggesting that most patients had satisfactory GIF (Table S1, Supplemental Digital Content 2, http://links.lww.com/JS9/B186, Fig. 2A). Sixteen patients (8.7%) showed obvious clinical symptoms of depression, while 27 patients (14.8%) showed obvious clinical symptoms of anxiety (GAD-7 and PHQ-9, ≥10). Nineteen (10.4%) patients complained about poor ability to work after surgery (WAI, <27.0), and 31 (16.9%) had concerns of their body image (BIS, ≥10). Eleven (6%) patients obtained scores that were more than half of the total score of the PTED-21 scale, while 153 (83.6%) patients scored more than half of the total score of SOC-9, indicating an excellent ability of these patients to cope with pressure (Table S2, Supplemental Digital Content 2, http://links.lww.com/JS9/B186, Fig. 2B). The results of the symptom part of EORTC QLQ-C30 showed that few patients had a score over the third quartile of all symptoms. The average insomnia score was the highest, compared to nausea and vomiting (26.6±28.3 vs. 9.9 ±17.5, *P*<0.001) (Table S3, Supplemental Digital Content 2, http://links.lww.com/JS9/B186, Fig. 2C). Similar findings were shown in the function part of EORTC QLQ-C30. Few patients scored below the first quartile in various functions. The average value of physical function was the highest. In contrast, the average value of emotional function was the lowest (90.1±13.9 vs. 77.6 ±22.1, *P*<0.001) (Table S4, Supplemental Digital Content 2, http://links.lww.com/JS9/B186, Fig. 2D).

### Correlations between mental stress, GIF, and clinicopathological characteristics

A nonparametric sum rank test was adopted to analyze differences in the scores of mental stress and GIFs among the clinicopathological items. In general, older age patients had fewer concerns regarding their body image (*P*=0.004) and worse ability to work (*P*=0.035); female patients had a poorer ability to respond to stress (*P*=0.021) and suffered more constipation (*P*=0.015). Patients with both physical and mental work had poorer ability to work (*P*=0.003) and more financial difficulty (*P*<0.001). Married patients had better performance on SOC-9 (*P*=0.021). The symptoms of diarrhea diminished gradually after surgery (*P*=0.043). Patients with postoperative complications experienced more severe GI symptoms (*P*=0.018 and *P*=0.031, respectively). Unexpectedly, features of tumor invasiveness did not significantly affect mental stress and GIF. The type of surgical procedure was the dominant factor affecting mental stress and GIF. Compared to RR, LR led to reduced mental stress and substantially better GIF (Table 3 and Table 4). Regarding RR, pancreaticoduodenectomy with or without pylorus-preservation led to more severe GI symptoms, compared to distal pancreatectomy with or without splenectomy (Table S5, Supplemental Digital Content 2, http://links.lww.com/JS9/B186).

**Table 3:** Correlations between various subscales and clinicopathological characters.

			Current age				Sex			Current marriage			Educational level			Work type				
		<25	25y≤,<45y	45y<,≤60y	>60	P	Male	Female	P	Unmarried	Married	P	Senior high school and below	University and postgraduate	P	Mental labor	Physical labor	Both	No work	P
PHQ-9		4.62 ± 4.47	4.57 ± 4.97	3.68 ± 3.36	4.52 ± 4.75	0.491	3.47 ± 4.26	4.87 ± 4.87	0.065	5.39 ± 5.22	4.25 ± 4.58	0.180	4.15 ± 4.81	4.87 ± 4.70	0.195	3.98 ± 4.26	5.34 ± 4.40	5.00 ± 5.74	6.25 ± 6.95	0.301
GAD-7		3.67 ± 5.38	3.81 ± 4.49	2.33 ± 3.19	2.63 ± 3.02	0.273	2.53 ± 3.44	3.70 ± 4.54	0.117	4.20 ± 5.12	3.17 ± 4.02	0.152	3.32 ± 4.36	3.51 ± 4.30	0.638	3.02 ± 3.73	4.38 ± 5.15	3.00 ± 4.30	4.45 ± 5.90	0.037^**^,^b^
SOC-9		48.00 ± 11.42	48.58 ± 12.96	54.03 ± 9.05	47.63 ± 10.18	0.088	51.93 ± 10.09	48.92 ± 12.59	0.194	45.43 ± 13.84	51.00 ± 11.17	0.021^*^	49.08 ± 12.59	50.20 ± 11.60	0.545	49.75 ± 11.3	47.97 ± 12.68	53.40 ± 12.70	49.95 ± 15.38	0.385
PTED-21		12.43 ± 17.53	11.14 ± 14.60	9.25 ± 10.59	15.38 ± 15.45	0.750	15.07 ± 16.35	9.75 ± 13.2	0.021^*^	10.70 ± 14.00	11.17 ± 14.28	0.585	11.25 ± 15.46	10.88 ± 12.96	0.370	9.28 ± 12.47	14.28 ± 14.76	11.5 ± 18.68	16.45 ± 18.82	0.210
BIS		6.95 ± 8.90	5.21 ± 6.21	3.10 ± 3.50	2.13 ± 3.04	0.004^**^,^b^	3.82 ± 5.16	5.16 ± 6.36	0.145	6.05 ± 7.42	4.45 ± 5.60	0.124	4.76 ± 5.71	4.89 ± 6.48	0.619	4.53 ± 5.43	5.66 ± 7.63	3.30 ± 5.06	6.10 ± 7.66	0.621
WAI		41.10 ± 6.56	39.11 ± 8.04	39.23 ± 7.41	39.23 ± 7.41	0.035^*^	37.62 ± 8.38	39.35 ± 8.13	0.109	40.43 ± 6.85	38.45 ± 8.56	0.197	40.06 ± 7.31	37.87 ± 8.87	0.104	40.64 ± 7.05	36.56 ± 9.34	34.3 ± 8.65	34.60 ± 9.70	0.003^*^
EORTC QLQ-C30	Fatigue	18.52 ± 16.60	21.83 ± 21.26	19.72 ± 22.15	45.83 ± 34.85	0.142	17.28 ± 19.90	23.59 ± 22.68	0.095	22.73 ± 22.09	21.82 ± 22.24	0.667	18.81 ± 18.94	25.03 ± 24.47	0.125	20.84 ± 21.21	24.65 ± 23.06	21.11 ± 19.91	25.56 ± 27.71	0.839
	Nausea and vomiting	8.73 ± 10.82	10.82 ± 17.46	7.50 ± 17.68	12.50 ± 19.42	0.371	7.04 ± 13.98	10.87 ± 18.42	0.240	8.71 ± 14.60	10.31 ± 18.32	0.930	9.09 ± 16.16	10.70 ± 18.66	0.640	9.37 ± 16.93	13.02 ± 19.28	8.33 ± 21.15	9.17 ± 16.64	0.580
	Pain	11.11 ± 16.10	14.33 ± 19.57	13.75 ± 20.98	35.42 ± 36.12	0.322	12.22 ± 21.44	15.58 ± 20.56	0.169	14.02 ± 17.59	14.99 ± 21.73	0.703	12.31 ± 17.96	17.02 ± 22.93	0.217	13.64 ± 19.84	20.83 ± 22.80	11.67 ± 22.29	13.33 ± 22.03	0.235
	Dyspnea	17.46 ± 22.65	19.01 ± 24.68	18.33 ± 21.28	29.17 ± 27.82	0.685	14.81 ± 21.97	20.53 ± 24.27	0.145	17.42 ± 24.37	19.66 ± 23.68	0.470	17.8 ± 22.00	20.35 ± 25.40	0.663	18.46 ± 23.94	23.96 ± 24.30	6.67 ± 14.05	21.67 ± 24.84	0.181
	Insomnia	14.29 ± 19.92	26.32 ± 28.21	31.67 ± 29.18	37.50 ± 37.53	0.111	20.74 ± 22.8	28.50 ± 29.75	0.187	24.24 ± 27.25	27.34 ± 28.73	0.550	23.11 ± 26.91	29.82 ± 29.36	0.107	24.52 ± 28.14	30.21 ± 22.97	20.00 ± 35.83	36.67 ± 32.26	0.120
	Appetite loss	7.94 ± 14.55	15.50 ± 24.76	7.50 ± 16.00	20.83 ± 24.80	0.179	8.89 ± 17.98	14.49 ± 23.47	0.179	12.88 ± 21.82	13.19 ± 22.56	0.990	14.02 ± 23.55	12.28 ± 21.22	0.696	13.22 ± 22.56	9.38 ± 17.42	10.00 ± 22.50	20.00 ± 27.36	0.507
	Constipation	31.75 ± 19.65	21.35 ± 26.65	22.50 ± 28.63	33.33 ± 25.20	0.074	14.81 ± 20.79	26.09 ± 27.53	0.015^*^	23.48 ± 24.46	23.26 ± 27.11	0.758	20.83 ± 26.41	25.61 ± 26.39	0.155	22.04 ± 26.72	29.17 ± 29.02	23.33 ± 27.44	21.67 ± 19.57	0.603
	Diarrhea	34.92 ± 28.82	27.78 ± 29.72	19.17 ± 21.20	8.33 ± 15.43	0.054	24.44 ± 23.99	26.33 ± 29.17	0.972	34.85 ± 29.60	23.02 ± 26.87	0.014^*^	28.03 ± 28.98	23.86 ± 26.92	0.345	25.34 ± 28.23	31.25 ± 28.23	23.33 ± 27.44	21.67 ± 24.84	0.640
	Financial difficulty	12.70 ± 26.82	11.11 ± 24.95	15.00 ± 23.81	29.17 ± 33.03	0.120	20.00 ± 29.64	10.63 ± 23.48	0.022^*^	7.58 ± 21.40	14.63 ± 26.35	0.053	8.33 ± 20.99	17.19 ± 28.29	0.008^**^	7.16 ± 18.36	15.63 ± 29.31	36.67 ± 36.68	31.67 ± 33.29	<0.001^*^
	Social function	93.65 ± 12.33	90.20 ± 19.68	87.50 ± 18.78	58.33 ± 38.83	0.032^*^	83.70 ± 23.16	90.22 ± 19.91	0.048^*^	91.67 ± 18.85	87.65 ± 21.45	0.233	91.1 ± 20.21	86.32 ± 21.33	0.033^*^	92.56 ± 15.51	87.50 ± 20.74	76.67 ± 32.58	72.50 ± 31.66	0.002^*^
	Cognitive function	84.13 ± 22.65	80.26 ± 20.87	83.33 ± 21.01	64.58 ± 27.37	0.153	84.81 ± 18.40	79.35 ± 22.38	0.187	80.30 ± 22.24	80.82 ± 21.41	0.966	81.06 ± 22.63	80.35 ± 20.63	0.567	83.2 ± 20.69	77.08 ± 19.28	70.00 ± 29.19	76.67 ± 24.42	0.115
	Emotional function	79.76 ± 22.60	74.71 ± 23.31	84.17 ± 17.38	79.17 ± 18.37	0.160	83.89 ± 16.13	75.48 ± 23.35	0.054	73.3 ± 25.01	78.90 ± 20.96	0.200	76.89 ± 24.26	78.16 ± 19.91	0.853	77.55 ± 21.30	78.65 ± 18.32	78.33 ± 25.22	75.42 ± 30.65	0.970
	Role function	91.27 ± 16.35	87.43 ± 18.76	86.67 ± 21.42	52.08 ± 33.85	0.004^**^	86.30 ± 21.11	86.11 ± 21.18	0.877	87.50 ± 19.73	85.73 ± 21.57	0.712	88.07 ± 17.86	84.39 ± 23.67	0.501	88.02 ± 19.15	81.25 ± 24.96	90.00 ± 14.05	80.83 ± 27.19	0.392
	Physical function	93.65 ± 9.30	91.64 ± 11.96	91.00 ± 10.08	65.00 ± 32.81	0.044^*^	91.11 ± 15.63	90.39 ± 13.29	0.443	90.91 ± 12.87	90.46 ± 13.86	0.802	92.88 ± 10.25	88.42 ± 16.28	0.067	91.96 ± 11.74	88.54 ± 15.54	89.33 ± 11.84	86.00 ± 21.62	0.343
	Global Health	81.35 ± 25.54	79.75 ± 22.03	83.13 ± 19.75	61.46 ± 33.61	0.234	79.07 ± 21.66	80.13 ± 23.17	0.451	82.01 ± 21.03	79.2 ± 23.30	0.692	80.87 ± 22.61	78.95 ± 22.96	0.488	81.61 ± 20.91	76.56 ± 25.53	73.33 ± 31.62	77.92 ± 24.37	0.738
GSRS	Abdominal pain	1.68 ± 0.83	1.64 ± 0.73	1.60 ± 0.91	2.08 ± 1.07	0.590	1.60 ± 0.79	1.67 ± 0.80	0.347	1.72 ± 0.78	1.63 ± 0.80	0.358	1.63 ± 0.78	1.68 ± 0.81	0.772	1.61 ± 0.74	1.82 ± 0.92	1.80 ± 1.01	1.60 ± 0.85	0.602
	Reflux	1.48 ± 0.70	1.64 ± 0.87	1.75 ± 0.88	1.94 ± 1.15	0.404	1.77 ± 0.86	1.63 ± 0.87	0.267	1.64 ± 0.70	1.67 ± 0.92	0.657	1.64 ± 0.93	1.68 ± 0.81	0.365	1.58 ± 0.81	1.64 ± 0.67	2.30 ± 1.60	1.88 ± 0.89	0.245
	Indigestion	2.24 ± 1.24	1.99 ± 1.02	1.82 ± 0.88	2.16 ± 0.93	0.500	1.73 ± 0.73	2.08 ± 1.08	0.084	2.20 ± 1.09	1.92 ± 0.99	0.070	1.97 ± 0.97	2.01 ± 1.06	0.828	1.89 ± 0.90	2.40 ± 1.40	2.03 ± 0.95	1.91 ± 0.92	0.446
	Diarrhea	2.13 ± 1.20	1.96 ± 1.13	2.09 ± 0.84	2.04 ± 1.69	0.302	1.92 ± 0.98	2.04 ± 1.14	0.738	2.17 ± 1.28	1.96 ± 1.04	0.284	1.90 ± 1.08	2.12 ± 1.12	0.129	1.92 ± 0.99	2.32 ± 1.47	2.23 ± 1.16	1.98 ± 1.03	0.487
	Constipation	2.00 ± 0.61	1.85 ± 0.89	2.04 ± 1.21	2.33 ± 0.98	0.264	1.64 ± 0.75	2.03 ± 0.99	0.012^*^	1.86 ± 0.74	1.95 ± 1.00	0.858	1.81 ± 0.95	2.05 ± 0.93	0.036^*^	1.82 ± 0.91	2.40 ± 1.11	2.13 ± 0.95	1.77 ± 0.67	0.041^*^
	Average scores	1.96 ± 0.79	1.84 ± 0.68	1.87 ± 0.66	2.13 ± 0.82	0.702	1.73 ± 0.59	1.92 ± 0.72	0.173	1.96 ± 0.71	1.85 ± 0.69	0.299	1.81 ± 0.67	1.93 ± 0.72	0.239	1.78 ± 0.65	2.17 ± 0.78	2.08 ± 0.69	1.83 ± 0.70	0.035^*^

* *P*-value <0.05.

** *P*-value <0.01;

b *P*-value was derived by the Pearson *χ* ^2^ test; the other *P*-values were derived by nonparametric sum rank test.

**Table 4:** Correlations between various subscales and surgical features.

	Postsurgery interval				Surgery procedures			Surgery methods			Pathology			surgery complications		
	<36 m	36≤，≤60 m	> 60m	*P*	LR	RR	*P*	Open	Minimally invasive	P	Non invasiveness	Invasiveness	*P*	Other	Clavien–Dindo 2 and beyond	*P*
PHQ-9	4.39±4.07	3.95±4.15	4.96±5.33	0.738	3.37±4.05	4.91±4.92	0.050^*^	4.84±5.08	4.22±4.43	0.513	4.62±4.68	4.16±5.07	0.341	4.61±4.68	4.26±5.04	0.419
GAD-7	4.09±4.70	3.27±3.61	3.27±4.62	0370	2.41±3.97	3.75±4.39	0.015^*^	3.31±4.27	3.51±4.38	0.664	3.60±4.41	2.71±3.90	0.203	3.49±4.56	3.19±3.45	0.673
SOC-9	21.64±11.74	21.9±11.64	22.89±12.56	0.880	20.52±10.65	22.95±12.48	0.371	22.6±12.34	22.10±11.86	0.860	22.43±12.09	21.97±12.13	0.688	23.14±12.62	19.74±9.74	0.210
PTED-21	13.73±15.04	9.42±12.72	11.18±14.77	0.171	7.93±9.55	12.11±15.31	0.287	10.97±13.64	11.15±14.74	0.617	11.20±13.83	10.53±15.63	0.412	10.56±13.76	12.7±15.50	0.373
BIS	5.36±5.88	4.77±5.67	4.68±6.51	0.541	3.61±5.07	5.24±6.38	0.093	4.75±6.07	4.90±6.16	0.665	4.67±6.11	5.45±6.13	0.326	4.82±6.22	4.86±5.78	0.969
WAI	40.33±5.87	38.07±8.81	38.98±8.52	0.763	41.83±6.3	37.95±8.55	0.006**	38.44±8.85	39.38±7.57	0.656	39.41±7.71	37.05±9.77	0.277	39.27±8.00	37.79±8.83	0.355
EORTC QLQ-C30																
Fatigue	23.57±20.56	21.48±21.15	21.85±23.52	0.614	12.32±14.86	25.3±23.25	0.001^**^	22.35±23.06	21.75±21.36	0.898	21.84±22.20	22.81±22.21	0.753	20.87±21.51	25.84±23.97	0.243
Nausea and vomiting	9.09±16.71	11.11±16.99	9.44±18.20	0.571	5.07±10.46	11.56±19.02	0.062	11.42±19.07	8.51±15.79	0.365	10.57±17.77	7.46±16.30	0.250	8.81±16.42	13.57±20.33	0.260
Pain	12.63±16.15	16.67±20.81	14.26±22.29	0.625	8.70±16.76	16.79±21.63	0.006^**^	16.1±23.36	13.48±18.01	0.952	14.48±19.86	15.79±24.18	0.908	14.52±20.37	15.5±22.24	0.816
Dyspnea	20.2±26.27	21.67±24.41	17.04±22.48	0.495	13.77±19.34	20.92±24.92	0.110	19.1±24.04	19.15±23.69	0.954	18.62±24.18	21.05±22.49	0.407	18.81±24.06	20.16±23.16	0.633
Insomnia	25.25±23.61	27.22±29.11	26.67±29.65	0.980	25.36±25.52	27.01±29.30	0.942	29.21±29.22	24.11±27.39	0.226	26.67±29.03	26.32±25.89	0.860	25.95±27.14	28.68±32.19	0.815
Appetite loss	14.14±23.61	13.33±19.60	12.59±23.74	0.673	7.25±20.98	15.09±22.50	0.008^**^	13.86±22.92	12.41±21.85	0.622	13.33±23.04	12.28±19.64	0.921	11.67±21.13	17.83±25.56	0.147
Constipation	22.22±25.91	18.33±25.62	27.04±26.86	0.088	21.74±27.41	23.84±26.18	0.522	24.72±27.77	21.99±25.19	0.589	22.99±26.21	24.56±27.60	0.781	22.86±25.94	24.81±28.26	0.769
Diarrhea	32.32±25.66	30.00±31.71	20.74±25.27	0.043^*^	23.91±26.91	26.52±28.34	0.616	25.84±28.32	25.89±27.72	0.941	24.83±27.15	29.82±30.79	0.403	26.19±28.77	24.81±25.30	0.972
Financial difficulty	12.12±26.11	13.33±24.70	12.96±25.82	0.834	6.52±19.40	15.09±26.8	0.029^*^	17.23±28.48	8.87±21.41	0.021^*^	11.72±23.74	17.54±30.74	0.280	12.86±25.78	13.18±24.28	0.714
Social	88.38±17.91	89.72±17.92	87.96±23.71	0.848	95.65±13.35	86.25±22.41	0.001^**^	85.96±23.82	91.13±17.4	0.120	88.85±20.98	87.72±20.75	0.673	89.64±19.74	85.27±24.18	0.216
Cognitive	81.82±19.70	80.00±20.54	80.74±23.02	0.858	85.51±21.26	79.08±21.49	0.037^*^	77.72±23.56	83.51±19.17	0.103	80.69±21.93	80.7±20.33	0.868	80.83±21.98	80.23±20.33	0.678
Emotional	73.23±19.63	78.19±21.76	78.7±23.11	0.201	81.70±21.92	76.16±22.01	0.079	77.15±23.18	77.93±21.05	0.988	76.67±21.82	80.92±22.92	0.126	77.8±22.12	76.74±22.09	0.678
Role	85.35±19.88	87.78±19.86	85.37±22.46	0.586	91.3±13.94	84.43±22.8	0.125	86.33±21.70	85.99±20.64	0.720	86.44±20.69	85.09±22.86	0.908	86.43±22.04	85.27±17.89	0.196
Physical function	91.72±10.81	90.56±11.86	90.15±16.03	0.744	95.36±6.42	88.95±15.26	0.026^*^	90.86±13.76	90.28±14.02	0.850	90.8±13.92	89.65±13.75	0.564	90.81±13.49	89.77±15.14	0.792
Global Health	80.30±21.23	77.5±22.62	81.3±23.48	0321	88.59±18.37	76.95±23.38	0.001^**^	80.24±22.87	79.52±22.75	0.749	80.69±22.54	76.75±23.58	0329	79.11±23.58	82.36±19.85	0.543
GSRS																
Abdominal pain	1.69±0.75	1.71±0.81	1.61±0.81	0.545	1.31±0.51	1.77±0.84	0.001^**^	1.69±0.89	1.62±0.70	0.731	1.65±0.79	1.66±0.84	0.730	1.63±0.78	1.76±0.87	0.076
Reflux	1.68±0.76	1.61±0.81	1.69±0.94	0.831	1.50±0.72	1.72±0.91	0.138	1.74±1.01	1.59±0.71	0.761	1.69±0.90	1.55±0.73	0.617	1.60±0.81	1.92±1.07	0.031^*^
Indigestion	1.77±0.77	2.24±1.13	1.90±0.99	0.077	1.76±0.86	2.07±1.05	0.081	2.09±1.05	1.90±0.98	0.290	2.01±1.00	1.92±1.07	0399	1.92±0.97	2.32±1.16	0.018^*^
Diarrhea	1.95±0.78	2.12±1.13	1.96±1.19	0.429	1.84±1.11	2.07±1.10	0.140	2.16±1.23	1.87±0.95	0.133	1.99±1.11	2.10±1.07	0.436	2.03±1.15	1.93±0.87	0.485
Constipation	1.72±0.78	1.79±0.87	2.10±1.02	0.083	1.91±1.09	1.94±0.90	0.407	2.05±1.02	1.82±0.86	0.153	1.95±0.97	1.85±0.86	0.697	1.93±0.97	1.95±0.86	0.514
Average scores	1.77±0.59	1.94±0.69	1.87±0.74	0.551	1.68±0.58	1.94±0.72	0.041^*^	1.97±0.74	1.78±0.64	0.104	1.88±0.70	1.84±0.68	0.794	1.84±0.69	2.00±0.73	0.054

* *P*-value <0.05.

** *P*-value <0.01; the *P*-values were derived by nonparametric sum rank test.

Multiple linear regression was used to analyze the correlation between mental stress, GIFs, and clinicopathological characteristics. As shown above, postoperative complications led to more severe GI symptoms. Diarrhea gradually diminished 5-years after surgery. MIS resulted in less postoperative GI dysfunction and better cognitive function (EORTC QLQ-C30, three subdomains, *P*<0.05; GSRS, two subdomains and the average score, *P*<0.05). LR led to less mental stress (PHQ-9, *P*=0.018), better ability to work (WAI, *P*=0.010), less GI dysfunction and better quality of life (EORTC QLQ-C30, nine subdomains, *P*<0.05; GSRS, three subdomains and the average score, *P*< 0.05) (Table 5) (Table 6).

**Table 5:** Multiple linear regression of subscales and clinicopathological characters.

					Work type^a^									
	Sex		Current marriage		Mental labor		Physical labor		Both		Current age		Educational level	
	*β*	*P*	*β*	*P*	*β*	*P*	*β*	*P*	*β*	*P*	*β*	*P*	*β*	*P*
PHQ-9	0.121	0.122	−0.124	0.168	−0.190	0.107	−0.055	0.615	−0.031	0.725	0.026	0.792	0.029	0.716
GAD-7	0.081	0.303	−0.009	0.923	−0.166	0.163	−0.014	0.898	−0.042	0.641	−0.139	0.156	0.011	0.890
SOC-9	−0.038	0.626	0.155	0.087	−0.021	0.856	−0.073	0.506	0.022	0.804	0.110	0.261	0.014	0.865
PTED-21	−0.190	0.016^*^	0.055	0.540	−0.253	0.032^*^	−0.066	0.541	−0.090	0.310	−0.121	0.212	−0.070	0.377
BIS	0.028	0.723	0.033	0.713	−0.103	0.387	−0.011	0.923	−0.073	0.413	−0.255	0.010^**^	0.049	0.540
WAI	0.020	0.790	−0.036	0.673	0.291	0.011^*^	0.057	0.583	0.007	0.936	−0.131	0.161	−0.002	0.976
EORTC QLQ -C30														
Fatigue	0.162	0.036^*^	−0.074	0.399	−0.018	0.877	0.025	0.813	−0.032	0.710	0.172	0.071	0.088	0.259
Nausea and vomiting	0.078	0.319	0.136	0.128	0.043	0.715	0.099	0.359	−0.009	0.918	−0.146	0.130	0.051	0.517
Pain	0.121	0.120	−0.035	0.692	0.085	0.470	0.170	0.118	−0.018	0.836	0.178	0.066	0.074	0.349
Dyspnea	0.130	0.102	0.039	0.666	−0.020	0.864	0.064	0.560	−0.139	0.122	0.100	0.308	0.038	0.636
Insomnia	0.181	0.022^*^	−0.020	0.825	−0.157	0.184	−0.071	0.517	−0.145	0.104	0.215	0.028^*^	0.070	0.375
Appetite loss	0.095	0.231	0.059	0.518	−0.140	0.240	−0.174	0.114	−0.091	0.311	−0.035	0.724	−0.055	0.491
Constipation	0.194	0.015^*^	0.013	0.885	0.053	0.654	0.143	0.196	0.032	0.723	−0.017	0.864	0.099	0.220
Diarrhea	−0.030	0.705	−0.061	0.499	0.058	0.625	0.126	0.247	0.049	0.578	−0.194	0.047^*^	−0.026	0.739
Financial difficulty	−0.089	0.219	0.078	0.352	−0.393	<0.001^**^	−0.222	0.029^*^	0.017	0.834	0.034	0.706	0.047	0.526
Social function	0.063	0.386	0.077	0.355	0.403	<0.001^**^	0.255	0.012^*^	0.073	0.373	−0.256	0.005^**^	0.049	0.503
Cognitive function	−0.150	0.055	0.034	0.702	0.120	0.306	−0.004	0.973	−0.089	0.313	−0.083	0.390	0.057	0.469
Emotional function	−0.141	0.078	0.042	0.643	0.054	0.648	0.066	0.551	−0.005	0.953	0.058	0.559	0.023	0.780
Role function	−0.077	0.324	0.092	0.305	0.120	0.305	−0.010	0.926	0.103	0.241	−0.313	0.001^**^	0.002	0.984
Physical function	−0.105	0.165	0.156	0.073	0.134	0.239	0.038	0.719	0.054	0.530	−0.363	<0.001^**^	−0.053	0.489
Global health	−0.014	0.858	−0.045	0.623	0.043	0.720	−0.045	0.679	−0.058	0.515	−0.040	0.683	0.018	0.823
GSRS														
Abdominal pain	0.052	0.504	−0.061	0.496	0.053	0.651	0.128	0.236	0.072	0.412	0.068	0.483	−0.020	0.799
Reflux	−0.044	0.575	−0.053	0.551	−0.156	0.186	−0.107	0.322	0.100	0.259	0.081	0.400	−0.065	0.409
Indigestion	0.137	0.074	−0.060	0.491	0.023	0.841	0.194	0.069	0.045	0.600	−0.030	0.749	0.005	0.944
Diarrhea	0.071	0.368	−0.085	0.344	0.039	0.740	0.135	0.216	0.061	0.493	0.040	0.680	0.079	0.324
Constipation	0.229	0.003^**^	0.014	0.873	0.075	0.516	0.280	0.009^**^	0.093	0.283	0.076	0.424	0.091	0.241
Average scores	0.143	0.062	−0.069	0.426	0.028	0.806	0.206	0.052	0.095	0.268	0.051	0.591	0.036	0.636

* *P*-value <0.05.

** *P*-value <0.01.

a Take ‘no work’ group as a reference to set the dummy variable.

**Table 6:** Multiple linear regression of subscales and surgical features of patients.

	Surgery method		Surgery procedure		Postsurgery interval		Pathology		Surgery complication	
	*β*	*P*	*β*	*P*	*β*	*P*	*β*	*P*	*β*	*P*
PHQ-9	−0.077	0.385	0.195	0.018^*^	0.100	0.278	−0.075	0.323	−0.020	0.783
GAD-7	−0.043	0.630	0.150	0.069	0.012	0.895	−0.112	0.143	−0.030	0.691
SOC-9	0.058	0.521	−0.134	0.103	−0.127	0.172	0.014	0.851	0.073	0.326
PTED-21	−0.028	0.754	0.143	0.080	0.015	0.873	−0.064	0.402	0.076	0.301
BIS	−0.064	0.475	0.104	0.208	0.010	0.910	0.037	0.633	0.009	0.903
WAI	0.078	0.363	−0.204	0.010^**^	0.063	0.472	−0.045	0.538	−0.073	0.306
EORTC QLQ-C30										
Fatigue	−0.099	0.257	0.283	0.001^**^	−0.026	0.773	−0.036	0.626	0.049	0.497
Nausea and vomiting	−0.204	0.023^*^	0.227	0.006^**^	−0.041	0.652	−0.113	0.136	0.075	0.312
Pain	−0.154	0.085	0.201	0.014^*^	−0.089	0.330	−0.008	0.917	−0.017	0.813
Dyspnea	−0.053	0.556	0.132	0.113	−0.040	0.665	0.023	0.762	0.005	0.950
Insomnia	−0.104	0.244	0.033	0.684	−0.088	0.338	−0.031	0.686	0.024	0.742
Appetite loss	−0.122	0.177	0.188	0.024^*^	−0.045	0.629	−0.077	0.314	0.096	0.199
Constipation	−0.028	0.759	0.056	0.496	0.091	0.328	0.039	0.612	−0.004	0.954
Diarrhea	−0.104	0.243	0.017	0.838	−0.105	0.256	0.070	0.360	−0.080	0.279
Financial difficulty	−0.221	0.008^**^	0.160	0.036^*^	−0.156	0.068	0.001	0.987	−0.011	0.870
Social function	0.132	0.111	−0.251	0.001^**^	0.023	0.787	0.076	0.278	−0.105	0.127
Cognitive function	0.192	0.032^*^	−0.191	0.020^*^	0.059	0.518	0.058	0.447	0.033	0.652
Emotional function	0.116	0.200	−0.140	0.092	0.057	0.539	0.116	0.135	0.031	0.683
Role function	0.007	0.938	−0.142	0.082	0.050	0.585	0.010	0.899	0.017	0.813
Physical function	−0.021	0.812	−0.204	0.010**	−0.001	0.987	0.012	0.866	−0.003	0.964
Global health	0.050	0.578	−0.232	0.005^**^	0.035	0.709	−0.023	0.763	0.071	0.344
GSRS										
Abdominal pain	−0.170	0.057	0.302	<0.001^**^	−0.070	0.445	−0.060	0.429	0.051	0.484
Reflux	−0.104	0.241	0.167	0.041^*^	−0.015	0.869	−0.129	0.089	0.134	0.070
Indigestion	−0.215	0.014^*^	0.176	0.028^*^	−0.110	0.219	−0.077	0.298	0.135	0.062
Diarrhea	−0.243	0.007^**^	0.116	0.158	−0.151	0.101	0.005	0.948	−0.058	0.433
Constipation	−0.087	0.318	0.066	0.412	0.057	0.529	−0.033	0.656	0.007	0.927
Average scores	−0.241	0.006^**^	0.220	0.006^*^	−0.094	0.292	−0.073	0.325	0.070	0.330

* *P*-value **<**0.05.

** *P*-value <0.01.

### Change of marital status after surgery

A total of 65 patients were unmarried when operated upon. Up to the last follow-up, 25 (38.5%) patients were married, and 40 (61.5%) were still unmarried. Four patients got divorced after surgery. More patients under 25 years old were in the unmarried population (*P*<0.001). Change in marital status was unrelated to other clinicopathological characteristics (Table S6, Supplemental Digital Content 2, http://links.lww.com/JS9/B186).

### Correlations between mental stress and GIF

The correlation between each GI symptom of GSRS and mental stress was analyzed next. This analysis revealed that poorer GI symptoms had a substantial negative impact on the patients’ mental stress and functions. Details were presented in Table 7.

**Table 7:** Correlations between psychological scales and GSRS.

	GSRS					
	Abdominal pain	Reflux	Indigestion	Diarrhea	Constipation	Average score
PHQ-9						
r	0.519	0.398	0.464	0.480	0.355	0.590
p	<0.001^**^	<0.001^**^	<0.001^**^	<0.001^**^	<0.001^**^	<0.001^**^
GAD-7						
r	0.405	0.370	0.361	0.320	0.289	0.475
p	<0.001^**^	<0.001^**^	<0.001^**^	<0.001^**^	<0.001^**^	<0.001^**^
SOC-9						
r	−0.345	−0.210	−0.319	−0.323	−0.224	−0.402
p	<0.001^**^	0.004^**^	<0.001^**^	<0.001^**^	0.002^**^	<0.001^**^
PTED-21						
r	0.426	0.407	0.327	0.306	0.193	0.443
p	<0.001^**^	<0.001^**^	<0.001^**^	<0.001^**^	0.009^**^	<0.001^**^
Function part of EORTC QLQ-C30						
Social function						
r	−0.394	−0.419	−0.308	−0.223	−0.124	−0.385
p	<0.001^**^	<0.001^**^	<0.001^**^	0.002^**^	0.094	<0.001^**^
Cognitive function						
r	−0.436	−0.393	−0.444	−0.405	−0.307	−0.550
p	<0.001^**^	<0.001^**^	<0.001^**^	<0.001^**^	<0.001^**^	<0.001^**^
Emotional function						
r	−0.420	−0.407	−0.415	−0.395	−0.293	−0.531
p	<0.001^**^	<0.001^**^	<0.001^**^	<0.001^**^	<0.001^**^	<0.001^**^
Role function						
r	−0.452	−0.285	−0.420	−0.216	−0.238	−0.448
p	<0.001^**^	<0.001^**^	<0.001^**^	<0.001^**^	<0.001^**^	<0.001^**^
Physical function						
r	−0.454	−0.363	−0.445	−0.329	−0.337	−0.534
p	<0.001^**^	<0.001^**^	<0.001^**^	<0.001^**^	<0.001^**^	<0.001^**^
Global health						
r	−0.380	−0.368	−0.368	−0.304	−0.212	−0.446
p	<0.001^**^	<0.001^**^	<0.001^**^	<0.001^**^	<0.001^**^	<0.001^**^

* *P*-value <0.05.

** *P*-value <0.01.

## Discussion

Increasing evidence, including our previous study, has largely alleviated concerns regarding tumor recurrence and metastasis of SPN after surgery, as LR has shown comparable oncological outcomes and morbidity rates to RR^3,4,7,22,23^. The predominant population of SPN patients comprises young and middle-aged women at a crucial period of their life and professional career. Diagnosing SPN and undergoing pancreatic surgery can potentially lead to significant mental stress and gastrointestinal dysfunction. However, no study has presented the postoperative mental stress and GIF of these patients, nor has there been any exploration of how to improve their postoperative GIF and reduce their mental stress through more precise and personalized surgical treatment strategies. In this study, we addressed these two issues by conducting a multiquestionnaire online survey in a long-term large cohort of SPN patients after surgery.

Furthermore, we innovated the assessment methods by using an online multiquestionnaire survey. We now show that 10–20% of patients had clinical symptoms of anxiety and depression after surgery, as demonstrated by poor performance on PHQ-9 and GAD-7. As indicated by WAI scores, ~10% of the patients complained of poor ability to work, and 16.9% of the patients had concerns about their body image, as indicated by BIS scores. In line, about 20% of the patients had GSRS scores above the median value. According to the EORTC QLQ-C30, the emotional function was most significantly affected compared to other functions. Mental stress and GIF correlated, indicating that more severe GI symptoms result in more mental stress. Multiple linear regression analyses showed that MIS resulted in fewer GI symptoms after surgery. It is reasonable to assume that patients with more aggressive tumors may have more severe mental stress; however, this could not be verified in the present analysis. It has to be pointed out that, since only a small proportion of patients had more aggressive tumors, this has to be interpreted carefully. Female patients complained of more severe emotional dysfunction and GI symptoms. Patients with physical work complained of more severe financial difficulties, and younger patients had more concerns regarding body image. Education level and marital status did not correlate with mental stress.

The main factor that affected mental stress and a GIF was LR. Thus, LR resulted in less mental stress and better GIF as compared to RR. The progress in pancreatic surgery and centralization in high-volume centers has made LR, including enucleation, duodenum preserving pancreatic head resection, and pancreatic segmental resection, safe and feasible^9,24,25^. During the last two decades, minimally invasive pancreatic surgery has rapidly progressed and robotic and laparoscopic-assisted pancreatic procedures have become routine in high-volume centers^26–28^. These advanced in organ preserving and minimally invasive methods enhance the recovery of the patients and reduce operative trauma^29,30^. Multilinear regression also showed that MIS led to fewer GI symptoms after surgery. Therefore, LR and MIS can potentially improve GIF and relieve the mental stress for SPN patients undergoing surgery.

### Limitations of this study

Due to the low incidence of SPN, conducting a randomized controlled study to explore the impact of LR and RR on postoperative mental stress and GIF is nearly impossible. While this study comprises a large cohort, there may be some selection biases that could potentially confound the results. In the future, external validations should be further conducted through multicenter studies in different regions and different countries to confirm the findings of this study. Additionally, being a cross-sectional study, it was unable to analyze dynamic changes in mental stress and GIF over time. Therefore, a prospective study would be needed to observe the dynamic changes in mental stress and GIF of SPN patients.

## Conclusion

This study presents, for the first time, data on the postoperative GIF and mental stress of SPN patients using a multiquestionnaire online survey in a large cohort from a high-volume center. The study findings demonstrate that LR and MIS significantly improve GIF and alleviate mental stress after surgery, in comparison to RR and open procedures. The results of this study could assist surgeons in devising more precise and personalized surgical plans for their patients.

## Ethics approval

This study was approved by the Institutional Review Board of PUMCH (SK1034) and registered at Clinicaltrial.org (NCT05604716) in November 2022.

## Consent

Before they answered the survey questions, they had to read the electronic written consent, and only those who agreed and confirmed the consent could access the survey.

## Sources of funding

This study was supported by the National High-Level Hospital Clinical Research Funding (2022-PUMCH-A-053, 2022-PUMCH-D-001), CAMS Innovation Fund for Medical Sciences (CIFMS, 2021-I2M-1-002), National Natural Science Foundation of China (52271254, 82172765, and 81872501), and Beijing Natural Science Foundation (Z190022): National Multidisciplinary Cooperative Diagnosis and Treatment Capacity Building Project for Major Diseases. The funding agencies did not participate in the design, data collection, interpretation, or manuscript writing.

## Author contribution

L.Q. (Qiaofei Liu), L.Q. (Quan Liao), H.Y., and H.X.: conceived and designed the study; Y.P. and W.W.: supervised this study; H.Y., L.J., H.X., Y.S., Z.H., and H.N.: implemented the online questionnaire, followed-up the patients, and collected and analyzed the data; Y.P., W.W., Z.T., D.M., G.J., W.W., H.X., and X.Q.: performed the operations; H.Y., L.Q. (Qiaofei Liu), L.J., and Y.S.: wrote the draft; J.K.: discussed the data and revised the draft; Y.P., W.W., and L.Q. (Quan Liao) revised the manuscript. All of the authors agreed to the content of this manuscript and to be listed as co-authors.

## Conflicts of interest disclosure

The authors declare that they have no known competing financial interests or personal relationships that could have appeared to influence the work reported in this paper.

## Research registration unique identifying number (UIN)

Registered at Clinicaltrial.org (NCT05604716) in November 2022. Cross-sectional Follow-up on Digestive and Social Functions of SPN - Full Text View- ClinicalTrials.gov.

## Guarantor

Prof. Qiaofei Liu.

## Data availability statement

All primary data are available from the corresponding author upon reasonable request.

## Provenance and peer review

Not commissioned, externally peer-reviewed.

## Supplementary Material

SUPPLEMENTARY MATERIAL

## References

[1] Dinarvand P Lai J. Solid pseudopapillary neoplasm of the pancreas: a rare entity with unique features. Arch Pathol Lab Med 2017;141:990–995. 28661210 10.5858/arpa.2016-0322-RS

[2] Stark A Donahue TR Reber HA. Pancreatic cyst disease: a review. JAMA 2016;315:1882–1893. 27139061 10.1001/jama.2016.4690

[3] Liu M Liu J Hu Q. Management of solid pseudopapillary neoplasms of pancreas: a single center experience of 243 consecutive patients. Pancreatology 2019;19:681–685. 31281058 10.1016/j.pan.2019.07.001

[4] Wright MJ Javed AA Saunders T. Surgical resection of 78 pancreatic solid pseudopapillary tumors: a 30-year single institutional experience. J Gastrointest Surg 2020;24:874–881. 31073801 10.1007/s11605-019-04252-7

[5] Law JK Ahmed A Singh VK. A systematic review of solid-pseudopapillary neoplasms: are these rare lesions? Pancreas 2014;43:331–337. 24622060 10.1097/MPA.0000000000000061 PMC4888067

[6] Kang CM Choi SH Kim SC. Predicting recurrence of pancreatic solid pseudopapillary tumors after surgical resection: a multicenter analysis in Korea. Ann Surg 2014;260:348–355. 24743622 10.1097/SLA.0000000000000583

[7] Zhan H Cheng Y Wang L. Clinicopathological features and treatment outcomes of solid pseudopapillary neoplasms of the pancreas: a 10-year case series from a single center. J Laparoendosc Adv Surg Tech A 2019;29:600–607. 30741591 10.1089/lap.2018.0704

[8] Estrella JS Li L Rashid A. Solid pseudopapillary neoplasm of the pancreas: clinicopathologic and survival analyses of 64 cases from a single institution. Am J Surg Pathol 2014;38:147–157. 24418850 10.1097/PAS.0000000000000141

[9] Tan C Yang Z Li J. Organ-preserving surgery and classic surgery for pancreatic solid pseudopapillary neoplasms: a multicenter analysis from central and western China. J Pancreatol. 2023;6:55–60.

[10] Liu Q Dai M Guo J. Long-term survival, quality of life, and molecular features of the patients with solid pseudopapillary neoplasm of the pancreas: a retrospective study of 454 cases. Ann Surg 2023. Online ahead of print. doi:10.1097/SLA.0000000000005842 37036095

[11] Tang L Fritzsche K Leonhart R. Emotional distress and dysfunctional illness perception are associated with low mental and physical quality of life in Chinese breast cancer patients. Health Qual Life Outcomes 2017;15:231. 29191208 10.1186/s12955-017-0803-9 PMC5709963

[12] Cloyd JM Tran Cao HS Petzel MQ. Impact of pancreatectomy on long-term patient-reported symptoms and quality of life in recurrence-free survivors of pancreatic and periampullary neoplasms. J Surg Oncol 2017;115:144–150. 27859270 10.1002/jso.24499 PMC11849053

[13] Allen CJ Yakoub D Macedo FI. Long-term quality of life and gastrointestinal functional outcomes after pancreaticoduodenectomy. Ann Surg 2018;268:657–664. 30199443 10.1097/SLA.0000000000002962 PMC7962861

[14] Dai M Liu Q Xing C. Early drain removal after major pancreatectomy reduces postoperative complications: a single-center, randomized, controlled trial. J Pancreatol 2020;3:93–100.

[15] Dai M Liu Q Xing C. Early drain removal is safe in patients with low or intermediate risk of pancreatic fistula after pancreaticoduodenectomy: a multicenter, randomized controlled trial. Ann Surg 2022;275:e307–e314. 34117153 10.1097/SLA.0000000000004992

[16] Kulich KR Madisch A Pacini F. Reliability and validity of the Gastrointestinal Symptom Rating Scale (GSRS) and Quality of Life in Reflux and Dyspepsia (QOLRAD) questionnaire in dyspepsia: a six-country study. Health Qual Life Outcomes 2008;6:12. 18237386 10.1186/1477-7525-6-12 PMC2276197

[17] Hayami M Seshimo A Miyake K. Effects of emptying function of remaining stomach on QOL in postgastrectomy patients. World J Surg 2012;36:373–378. 22173591 10.1007/s00268-011-1379-x

[18] Okada K Kawai M Uesaka K. Effect of Daikenchuto (TJ-100) on postoperative bowel motility and on prevention of paralytic ileus after pancreaticoduodenectomy: a multicenter, randomized, placebo-controlled phase II trial (the JAPAN-PD study). Jpn J Clin Oncol 2013;43:436–438. 23365113 10.1093/jjco/hyt005

[19] Yoo HK Patel N Joo S. Health-related quality of life of patients with metastatic pancreatic cancer: a systematic literature review. Cancer Manag Res 2022;14:3383–3403. 36510575 10.2147/CMAR.S376261 PMC9738117

[20] Wang X Gao Y Tan L. Reliability and validity of the Chinese version of the post-traumatic embitterment disorder self-rating scale (PTED-21) among inpatients in general hospital. Clin Psychol Psychother 2021;28:882–890. 33338313 10.1002/cpp.2542

[21] Mathew G Agha R Albrecht J. STROCSS 2021: strengthening the reporting of cohort, cross-sectional and case-control studies in surgery. Int J Surg 2021;96:106165. 34774726 10.1016/j.ijsu.2021.106165

[22] Lee DH Han Y Byun Y. Central pancreatectomy versus distal pancreatectomy and pancreaticoduodenectomy for benign and low-grade malignant neoplasms: a retrospective and propensity score-matched study with long-term functional outcomes and pancreas volumetry. Ann Surg Oncol 2020;27:1215–1224. 31898101 10.1245/s10434-019-08095-z

[23] Yang F Wu W Wang X. Grading solid pseudopapillary tumors of the pancreas: the fudan prognostic index. Ann Surg Oncol 2021;28:550–559. 32424583 10.1245/s10434-020-08626-z

[24] Wujimaimaiti N Wu Y Yuan J. Laparoscopic duodenum-preserving pancreatic head resection: a narrative review. J Pancreatol 2021;4:146–152.

[25] Goudard Y Gaujoux S Dokmak S. Reappraisal of central pancreatectomy a 12-year single-center experience. JAMA Surg 2014;149:356–363. 24740703 10.1001/jamasurg.2013.4146

[26] Shi Y Wang W Qiu W. Learning curve from 450 cases of robot-assisted pancreaticoduocectomy in a high-volume pancreatic center: optimization of operative procedure and a retrospective study. Ann Surg 2021;274:e1277–e1283. 31651533 10.1097/SLA.0000000000003664

[27] Kim SC Song KB Jung YS. Short-term clinical outcomes for 100 consecutive cases of laparoscopic pylorus-preserving pancreatoduodenectomy: improvement with surgical experience. Surg Endosc 2013;27:95–103. 22752284 10.1007/s00464-012-2427-9

[28] Chen S Zhan Q Chen JZ. Robotic approach improves spleen-preserving rate and shortens postoperative hospital stay of laparoscopic distal pancreatectomy: a matched cohort study. Surg Endosc 2015;29:3507–3518. 25791063 10.1007/s00464-015-4101-5

[29] Dokmak S Fteriche FS Aussilhou B. The largest european single-center experience: 300 laparoscopic pancreatic resections. J Am Coll Surg 2017;225:226–234 e222. 28414116 10.1016/j.jamcollsurg.2017.04.004

[30] Xu Q Liu T Zou X. The learning curve for robot-assisted distal pancreatectomy: a single-center experience of 301 cases. J Pancreatol 2022;5:118–124. 36419868 10.1097/JP9.0000000000000096 PMC9665946

