# Supplementary material for: Local resection for solid pseudopapillary neoplasms of the pancreas shows improved postoperative gastrointestinal function and reduced mental stress: a multiquestionnaire survey from a large cohort
Source: Int J Surg. 2023 Oct 11;109(12):3815–26. doi: 10.1097/JS9.0000000000000702 (PMC10720864; doi:10.1097/JS9.0000000000000702)
Supplement: SUPPLEMENTARY MATERIAL [file js9-109-3815-s002.docx]

Table S1 Statistical description of GSRS

|  |  | Abdominal pain | Reflux | Indigestion | Diarrhea | Constipation | Average score |
| --- | --- | --- | --- | --- | --- | --- | --- |
| n |  | 183.0 | 183.0 | 183.0 | 183.0 | 183.0 | 183.0 |
| Average |  | 1.7 | 1.7 | 2.0 | 2.0 | 1.9 | 1.9 |
| Median |  | 1.3 | 1.5 | 1.8 | 1.7 | 1.7 | 1.7 |
| Standard deviation |  | 0.8 | 0.9 | 1.0 | 1.1 | 0.9 | 0.7 |
| Minimum value |  | 1.0 | 1.0 | 1.0 | 1.0 | 1.0 | 1.0 |
| Maximum value |  | 5.0 | 5.5 | 6.0 | 7.0 | 6.0 | 4.0 |
| Percentiles | 25 | 1.0 | 1.0 | 1.3 | 1.0 | 1.0 | 1.3 |
|  | 50 | 1.3 | 1.5 | 1.8 | 1.7 | 1.7 | 1.7 |
|  | 75 | 2.0 | 2.0 | 2.5 | 2.3 | 2.3 | 2.3 |

Table S2 Statistical description of SPFs, body image anxiety, and workability

|  |  | PHQ-9 | GAD-7 | SOC-9 | PTED-21 | BIS | WAI |
| --- | --- | --- | --- | --- | --- | --- | --- |
| n |  | 183 | 183 | 183 | 183 | 183 | 183 |
| Average |  | 4.5 | 3.4 | 49.7 | 11.1 | 4.8 | 38.9 |
| Median |  | 3.0 | 2.0 | 53.0 | 5.0 | 3.0 | 41.0 |
| Standard deviation |  | 4.8 | 4.3 | 12.1 | 14.2 | 6.1 | 8.2 |
| Minimum value |  | 0.0 | 0.0 | 10.0 | 0.0 | 0.0 | 10.0 |
| Maximum value |  | 24.0 | 21.0 | 63.0 | 67.0 | 28.0 | 49.0 |
| Percetiles | 25 | 0.0 | 0.0 | 42.0 | 1.0 | 1.0 | 34.0 |
|  | 50 | 3.0 | 2.0 | 53.0 | 5.0 | 3.0 | 41.0 |
|  | 75 | 7.0 | 5.0 | 60.0 | 16.0 | 7.0 | 45.0 |

Table S3 Statistic description of symptom part of EORTC-QLQ C30

|  |  | Fatigue | Nausea and vomiting | Pain | Dyspnea | Insomnia | Appetite loss | Constipation | Diarrhea | Financial difficulty |
| --- | --- | --- | --- | --- | --- | --- | --- | --- | --- | --- |
| n |  | 183.0 | 183.0 | 183.0 | 183.0 | 183.0 | 183.0 | 183.0 | 183.0 | 183.0 |
| Average |  | 22.0 | 9.9 | 14.8 | 19.1 | 26.6 | 13.1 | 23.3 | 25.9 | 12.9 |
| Median |  | 22.2 | 0.0 | 0.0 | 0.0 | 33.3 | 0.0 | 33.3 | 33.3 | 0.0 |
| Standard deviation |  | 22.1 | 17.5 | 20.8 | 23.8 | 28.3 | 223 | 26.4 | 27.9 | 254 |
| Minimum value |  | 0.0 | 0.0 | 0.0 | 0.0 | 0.0 | 0.0 | 0.0 | 0.0 | 0.0 |
| Maximum value |  | 100.0 | 833 | 100.0 | 100.0 | 100.0 | 100.0 | 100.0 | 100.0 | 100.0 |
| Percentiles | 25.0 | 0.0 | 0.0 | 0.0 | 0.0 | 0.0 | 0.0 | 0.0 | 0.0 | 0.0 |
|  | 50.0 | 222 | 0.0 | 0.0 | 0.0 | 33.3 | 0.0 | 33.3 | 33.3 | 0.0 |
|  | 75 | 333 | 16.7 | 16.7 | 333 | 33.3 | 333 | 33.3 | 33.3 | 333 |

Table S4 Statistic description of function part of EORTC-QLQ C30

|  |  | Social function | Cognitive function | Emotional function | Role function | Physical function | Global Health |
| --- | --- | --- | --- | --- | --- | --- | --- |
| N |  | 183.0 | 183.0 | 183.0 | 183.0 | 183.0 | 183.0 |
| Average |  | 88.6 | 80.7 | 77.6 | 86.2 | 90.6 | 79.9 |
| Median |  | 100.0 | 83.3 | 83.3 | 100.0 | 100.0 | 83.3 |
| Standard deviation |  | 20.9 | 21.6 | 22.1 | 21.1 | 13.9 | 22.8 |
| Minimum value |  | 0.0 | 0.0 | 0.0 | 0.0 | 20.0 | 0.0 |
| Maximum value |  | 100.0 | 100.0 | 100.0 | 100.0 | 100.0 | 100.0 |
| Percentiles | 25.0 | 83.3 | 66.7 | 66.7 | 66.7 | 86.7 | 66.7 |
|  | 50.0 | 100.0 | 83.3 | 83.3 | 100.0 | 100.0 | 83.3 |
|  | 75.0 | 100 | 100 | 100 | 100 | 100 | 100 |

Table S5 Differences of mental stress and GIF between PD and DP

|  | | **PD & PPPD** | **DP &including DPS** | **P** |
| --- | --- | --- | --- | --- |
| **n** |  | 33 | 104 | — |
| **PHQ-9** |  | 4.76±4.76 | 4.96±4.99 | 0.974 |
| **GAD-7** |  | 3.58±3.69 | 3.81±4.60 | 0.864 |
| **SOC-9** |  | 50.85±12.51 | 48.48±12.48 | 0.268 |
| **PTED-21** |  | 14.88±18.51 | 11.23±14.13 | 0.243 |
| **BIS** |  | 6.00±6.85 | 5.00±6.24 | 0.597 |
| **WAI** |  | 36.42±9.67 | 38.43±8.16 | 0.378 |
| **EORTC QLQ-C30** | **Fatigue** | 28.28±23.59 | 24.36±23.18 | 0.35 |
|  | **Nausea and vomiting** | 17.68±26.00 | 9.62±15.87 | 0.217 |
|  | **Pain** | 21.21±25.44 | 15.38±20.21 | 0.344 |
|  | **Dyspnea** | 23.23±25.66 | 20.19±24.76 | 0.524 |
|  | **Insomnia** | 24.24±29.19 | 27.88±29.42 | 0.494 |
|  | **Appetite loss** | 21.21±24.75 | 13.14±21.50 | 0.062 |
|  | **Constipation** | 15.15±23.70 | 26.6±26.43 | 0.018* |
|  | **Diarrhea** | 37.37±29.76 | 23.08±27.11 | 0.011* |
|  | **Financial difficulty** | 28.28±31.32 | 10.90±23.88 | ＜0.001** |
|  | **Social** | 78.28±25.17 | 88.78±20.96 | 0.004** |
|  | **Cognitive** | 76.26±21.66 | 79.97±21.46 | 0.312 |
|  | **Emotional** | 75.25±22.49 | 76.44±21.95 | 0.766 |
|  | **Role** | 79.80±24.92 | 85.90±22.00 | 0.125 |
|  | **Physical function** | 89.29±11.90 | 88.85±16.24 | 0.466 |
|  | **Global Health** | 76.01±25.24 | 77.24±22.88 | 0.988 |
| **GSRS** | **Abdominal pain** | 2.10±1.09 | 1.66±0.72 | 0.077 |
|  | **Reflux** | 1.85±1.14 | 1.67±0.82 | 0.793 |
|  | **Indigestion** | 2.45±1.22 | 1.95±0.97 | 0.044* |
|  | **Diarrhea** | 2.53±1.14 | 1.93±1.05 | 0.002** |
|  | **Constipation** | 1.80±0.87 | 1.98±0.90 | 0.25 |
|  | **Average scores** | 2.18±0.82 | 1.86±0.67 | 0.039* |

* P value＜0.05

** P value＜0.01

PD： pancreaticoduodenectomy

PPPD：pylorus-preserving pancreaticoduodenectomy

DP: distal pancreatectomy

DPS: distal pancreatectomy with splenectomy

|  |  | **Currently unmarried** | **Currently married** | **P** |
| --- | --- | --- | --- | --- |
| **Age** | <25y | 21 | 1 | 0.001^**^ |
|  | 25y≤, <45y | 19 | 24 |  |
|  | 45y≤, ≤60y | 0 | 0 |  |
|  | >60y | 0 | 0 |  |
| **Gender** | Male | 4 | 3 | 0.800 |
|  | Female | 36 | 22 |  |
| **Educational level** | Senior high school and below | 24 | 13 | 0.526 |
|  | University and postgraduate | 16 | 12 |  |
| **Work type** | Mental work | 27 | 16 | 0.056 |
|  | Physical labor | 0 | 3 |  |
|  | Both | 0 | 3 |  |
| **Post-surgery interval** | <36 m | 12 | 4 | 0.219 |
|  | 36≤，≤60 m | 14 | 7 |  |
|  | >60 m | 14 | 14 |  |
| **Surgery procedure** | LR | 10 | 6 | 0.927 |
|  | RR | 30 | 19 |  |
| **Surgical method** | Open | 17 | 14 | 0.289 |
|  | Minimal invasiveness | 23 | 11 |  |
| **Pathology** | Invasiveness | 9 | 6 | 0.889 |
|  | Non- invasiveness | 31 | 19 |  |
| **Surgical complications** | Clavien-Dindo 2 and beyond | 9 | 8 | 0.397 |
|  | Other | 31 | 17 |  |

Table S6 Correlation between marriage and clinicopathological and surgical features

** P value＜0.01
